# The Separation of Oil/Water Mixtures by Modified Melamine and Polyurethane Foams: A Review

**DOI:** 10.3390/polym13234142

**Published:** 2021-11-27

**Authors:** Sarah Mohammed Hailan, Deepalekshmi Ponnamma, Igor Krupa

**Affiliations:** Center for Advanced Materials, Qatar University, Doha P.O. Box 2713, Qatar; sarah.hailan@qu.edu.qa (S.M.H.); deepalekshmi@qu.edu.qa (D.P.)

**Keywords:** melamine, polyurethane, foams, emulsion separation, filtration, oil/water separation

## Abstract

Melamine (MA) and polyurethane (PU) foams, including both commercial sponges for daily use as well as newly synthesized foams are known for their high sorption ability of both polar and unipolar liquids. From this reason, commercial sponges are widely used for cleaning as they absorb a large amount of water, oil as well as their mixtures. These sponges do not preferentially absorb any of those components due to their balanced wettability. On the other hand, chemical and physical modifications of outer surfaces or in the bulk of the foams can significantly change their original wettability. These treatments ensure a suitable wettability of foams needed for an efficient water/oil or oil/water separation. MA and PU foams, dependently on the treatment, can be designed for both types of separations. The particular focus of this review is dealt with the separation of oil contaminants dispersed in water of various composition, however, an opposite case, namely a separation of water content from continuous oily phase is also discussed in some extent. In the former case, water is dominant, continuous phase and oil is dispersed within it at various concentrations, dependently on the source of polluted water. For example, waste waters associated with a crude oil, gas, shale gas extraction and oil refineries consist of oily impurities in the range from tens to thousands ppm [mg/L]. The efficient materials for preferential oil sorption should display significantly high hydrophobicity and oleophilicity and vice versa. This review is dealt with the various modifications of MA and PU foams for separating both oil in water and water in oil mixtures by identifying the chemical composition, porosity, morphology, and crosslinking parameters of the materials. Different functionalization strategies and modifications including the surface grafting with various functional species or by adding various nanomaterials in manipulating the surface properties and wettability are thoroughly reviewed. Despite the laboratory tests proved a multiply reuse of the foams, industrial applications are limited due to fouling problems, longer cleaning protocols and mechanical damages during performance cycles. Various strategies were proposed to resolve those bottlenecks, and they are also reviewed in this study.

## 1. Introduction

The Oil and grease are organic substances composed by different hydrocarbons, soaps, fatty acids, and waxes [1]. Petroleum wastewaters formed during a production and processing of crude oil, gas, shale gas extraction and oil refineries, food and metal processing waters mostly contribute to the formation of oily polluted waters [1]. The volumes of produced water were 202 billion barrels in 2014 and around 340 billion barrels in 2020 [2]. Emulsification of oil often occurs in water treatment processes due to the intensive use of emulsifiers, surfactants, and polymeric additives in petrochemical industry, mainly in the processes of chemically enhanced oil recovery of bypass oil and oil trapped in porous reservoirs [3,4]. These processes result in the production of huge volumes of oil-emulsified wastewater what implies serious problems of efficient water cleaning associated with a relatively high stability of emulsified mixtures and small particles size (below 5 microns) of oily droplets. Emulsified wastewater can be arbitrarily defined as a mixture of oil in water, mostly stabilized by various surfactants, having an average size of oily droplets below 20 μm [5,6,7]. Another definition says that the size of droplets within emulsions is in the range from 0.1 to 10 μm, what is associated with the range of visible light [8]. Such emulsions, together with truly dissolved organic hydrocarbons such as benzene, toluene, ethylbenzene, and xylene mean major challenges for water treatment, particularly for tertiary treatment of produced water. Water treated in such ways should not consist of more than 5 ppm of oily components in the outlet in order to respect most restrictive regulations for a water discharge into environment or its use for irrigation [1]. A few reviews focused on various aspects of materials, technologies, and theoretical modelling of tertiary treatment of produced water have been published very recently [9,10].

Since the complex configuration of the surfactant stabilized water/oil emulsions causes their separation a very complicated process, it is very necessary to explore the nature of emulsions and the strategies required to resolve their separation related issues. Generally, the oil-in-water and water-in-oil emulsions are very common in this intense mix of oil and water [10,11,12]. The floatation techniques such as dissolved air floatation, dispersed air floatation and electroflotation techniques are utilized for separating the oil/water emulsions, but numerous problems are associated with those methods [13]. High maintenance and running costs, difficulty to optimize the conditions depending on the nature of emulsions, and formation of gas bubbles during the process are very challenging. Thermo/chemical and electrolytic demulsification strategies are also followed for separating the complex emulsions [14], however, selective permeation materials based on foams [15], sponges [16], carbon materials [17] and porous membranes [18] are also reported for separating oil/water emulsions. Though the materials are notable in tuning the pore size, surface functionalities and wetting behavior, high cost, thickness of materials, weak mechanical properties and low efficiencies sometimes negatively influence their outstanding performance [15]. 

Superhydrophobic/superoleophilic materials are developed with opposite wettability to separate oil/water mixtures [19]. Coated steel meshes and cotton fabrics were the first used materials to separate oil from contaminated water but the high oil affinity of such materials caused biofouling and sometimes blocking of the pores [7]. Such problems affect the separation efficiency and therefore superhydrophilic/underwater superoleophobic steel meshes are designed, which further requires pre-wetting in water environment [20]. Several materials have been proposed for a separation of immiscible oil/water mixtures using superoleophobic/superhydrophilic steel meshes, fabrics and polymeric materials [21]. Structural strength, manufacturability and multifunctionality of polymers are preferable for manufacturing membranes for oil/water separation with some cases demanding surface modification for hydrophobic/hydrophilic properties [22]. Both phase inversion methods involving complex physical-chemical processes and in situ elimination methods are applied for generating porous polymeric membranes, which is much helpful in selectively separating oil/water mixtures and emulsions [12,23]. Micro/nano particles are also included in various matrices to develop additional characteristics such as mechanical strength, antibacterial properties and pollutant degradability [24].

PU as well as MA foams are well-known, cheap, and easily available 3D porous materials with continuous network structure and high surface area and have been extensively referred as very suitable substrates for additional treatment to get materials with tailored surface wettability. High elasticity and mechanical durability coupled with possible use of recycled products make them very good option as oil absorbing materials [25,26]. However, the natural wetting properties of these materials demand various types of physical and chemical modifications on their structure to strengthen the super hydrophobicity or super oleophilicity. Though significant research studies are focusing on functionalization techniques, binding with nanostructured materials, chemical etching and/or polymer grafting processes large scale contamination still suffers efficient, robust, and scalable methods of separation strategies. 

This review paper aims to summarize and evaluate recent findings focused on the MA and PU foams treatment, which are applicable for a separation of oil/water emulsions. Various modifications performed on the MA and PU foams in regulating the separation capabilities of the materials are discussed by emphasizing the following aspects: (i) characteristic properties of the foams and their dependence on the oil separating strategies, (ii) nature of oil/water emulsions and its influence on the material performance, (iii) commercial preferences and how the goals can be achieved by modification methods and (iv) different challenges during the development of advanced foam separators. 

## 2. Characterizations of the Foams

A typical oil/water emulsion separating foam must highly possess oil absorbing ability, fast absorption rate, and longer oil retention time and therefore modification of its wettability is a crucial point. Neat, untreated PU and MA foams are both hydrophilic and oleophobic, with high sorption capability for oil, water, and their mixtures. The separation of oil from oil in water mixtures requires enhanced oleophilicity and reduced hydrophilicity (enhanced hydrophobicity). Since cost effectiveness is an important factor for large-scale applications, for instance for the separations needed in petroleum industry, it is often desirable using available commercially foams instead of newly synthesized materials. In addition, the foam is expected to float on the water surface after the absorption of the oil and should be reusable [27]. Two major parameters that determine the capability of the polymeric foams in absorbing the oil from an emulsion are its efficiency and selectivity. While the emulsion separation efficiency of a polymeric foam is described by its ability to absorb oil over water and retain it, the selectivity attitude depends on the ability to selectively target the oil molecules. 

The wettability is a very important parameter in emulsion separation process as it is the prime factor in checking the efficiency of oil absorption. Wettability of solid materials is dictated by surface chemistry involving both natural chemical composition of outer surfaces, and additional chemical and physical surface post-treatment as well as by the topology (given by a roughness) of outer surfaces. Wettability of surfaces is commonly determined from the measurement of contact angles of droplets of various polar and nonpolar liquids placed on the top surface area. There exist at least three phases there, which play a role in a solid surface wettability: (i). solid surface, characterized by the surface tension, (ii). testing liquids characterized by the surface free energies, and the surroundings the phase which is in an intimate contact with both former phases. The third phase is often air, which has the surface energy equals zero. In the case of oil/water separation, the third phase is water of various composition, which interacts with both solid surface (a foam in this case) and dispersed oil contaminants. From this reason, underwater contact angle determination is a crucial point for an estimation of solid surface wettability by oil phase [28,29,30]. Considering a situation above, namely the determination of contact angle of oil on solid surface measured under water, the contact angle can be expressed by Equations (1)–(3) [31]:

(1) $${\cos\Theta }_{W}=\frac{\gamma_{\mathrm{SW}}{-\gamma }_{\mathrm{SA}}}{\gamma_{\mathrm{WA}}}$$

(2) $${\cos\Theta }_{O}=\frac{\gamma_{\mathrm{SO}}{-\gamma }_{\mathrm{SA}}}{\gamma_{\mathrm{OA}}}$$

(3) $${\;\cos\Theta }_{\mathrm{OW}}=\frac{\gamma_{\mathrm{OA}}{\cos\Theta }_{O}{-\gamma }_{\mathrm{WA}}{\cos\Theta }_{W}}{\gamma_{\mathrm{OW}}}$$

where γ_OA_, γ_OW_, and γ_WA_ are the oil/air, oil/water, and water/air interface tensions, respectively, and Θ_O_, Θ_OW_, and Θ_W_ are the contact angles of oil in air, oil in water, and water in air, respectively. It is evident from Equations (1)–(3) that a hydrophilic surfaces in air surroundings behave also as oleophilic ones in air because γ_OA_ << γ_WA_, and hydrophilic surfaces in air act as oleophobic ones in water, as can be seen from Equation (3) [31].

The wettability of rough surfaces is most frequently expressed by equations Equations (4) and (5) which describe the Wenzel and Cassie states.

(4) $${\cos\Theta }_{\mathrm{OW}}^{*}=r_{w}{\cos\Theta }_{\mathrm{OW}}$$

(5) $${\cos\Theta }_{\mathrm{OW}}^{*}={\mathrm{fcos}\Theta }_{\mathrm{OW}}+f-1$$

where Θ_OW_ and Θ_OW_^*^ are the contact angles of the oil droplet on the smooth and rough surface, respectively, in the water phase. The Wenzel state corresponds to the situation where an oil droplet is attached to a rough, superhydrophobic surface having the pores filled with air, and capillary forces can absorb oil into the pores what lead to the fully wetted surface by oil [32]. In the case of Cassie state, the valleys formed on the surface are filled by water, and therefore the penetration of oil into those cavities is suppressed, and oil droplets stay only on the ‘pin’ objects resulting in enhanced oleophobicity of those surfaces [31]. From previous remarks is evident that superhydrophobic and superoleophilic character of solid surfaces is given by the combination of the chemical composition and the controlled roughness [33].

**Scheme 1:**
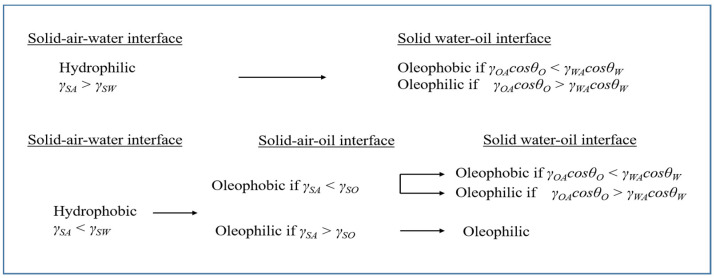
Summary of wettability characteristics of various interfaces.

The summary of the surface wettability regarding the nature of various interfaces is presented in Scheme 1 [34].

Surface properties are affected by the surface energy between the solid foam and the liquid in contact to it and by the roughness of the foam surface and its charge. The strong oxygen-hydrogen covalent bond of water creates high surface tension for water (72.8 mN/m) [35] while the oil’s surface tension is generally lower than that of water as it contains weaker bonds. Therefore, the polymeric foam surface energy should be in between the surface tension of oil and surface tension of water and in this case, the foam will selectively absorb oil and repel water, as there will be lower difference in the energy between oil and foam surface [35,36,37,38]. Indeed, suitable surface roughness should be optimized as it will provide capillary forces that prevent water from entering the grooves on the foam surface [39]. Also, residual charges in the surface can enhance the wetting performance of the foams. For example, a foam surface containing negatively charged groups or ions such as fluorine will repel its counterpart hydroxyl ions of water, thereby enhancing the hydrophobicity [38].

In addition to the surface topology, internal structure also directly affects the wetting performance of foams. This by means include the number of pores presented, their size and the interconnectivity between the pores. It is well established that higher porous foams will have higher oil capacity. This is because there is a clear correlation between the amount of gaseous phase in the foam and the volume of the absorbed oil. To fill the entire gaseous phase of the foam with oil, the capillary forces must be high enough to drive the oil absorption and retention. Indeed, the foam’s pores should be interconnected so the oil can penetrate and fill the total available volume in the foam. The capillary force depends on the diameter of the foam; therefore, by considering the oil properties (viscosity and surface tension) to the foam’s pore size, a high oil absorption capacity is achieved with maximum rate and longer retention. It should be noted that the optimum oil intake of PU foam is performed when the pore size is equal to or less than 500 μm [39]. In addition to pore size, the oil intake rate is affected by the overall pore connectivity and the tortuosity of the porous structure. As the pores connectivity approaches 100%, the rate of oil intake increases dramatically. Conversely, the tortuosity of the porous structure devalues the oil absorption rate as it creates longer path for the oil to get into the inner pores. Pinto et al. correlated the absorption capacity (*AC*) and the foam’s porosity by the Equation (6) [40]:

(6) $$AC=V_{f}.\frac{\rho_{oil}}{\rho_{f}}$$

where $\rho_{f}$ is the foam’s density and $\rho_{oil}$ oil’s density. The oil intake/capacity was measured by weighting the polymeric foam before and after certain time of contact with the liquid [40]. Ideally, a hydrophobic foam does not absorb water at all, however, in reality, the foam might show some sticky water contact on its surface and thus by measuring the water intake, the hydrophobicity of the foam is determined. Similarly, oil absorption capacity is determined by measuring the weight of the polymeric foam after certain time of contact with oil [41]. Thus, for oil/water separation applications, the polymeric foam with highest oil intake and lowest water intake is always targeted.

Functionalization/modification of commercially available polymeric foams is advantageous than synthesizing specially designed complex materials from an economical point of view. This is often done by the addition of organic compounds via different deposition techniques. The needed wetting property is also obtained through coating the foam’s surface with hydrophobic and/or oleophilic materials. Since reusability and durability are key advantages of foams compared to other sorbent materials, exploring these parameters depending on the quality of treatment is very significant. Normally the cleanup process for foam is done by simply squeezing by support vessel, extraction in hot water or hot steam and/or extraction by organic solvents, which is the least feasible method [42].

### 2.1. MA Based Foams 

**Figure 1:**
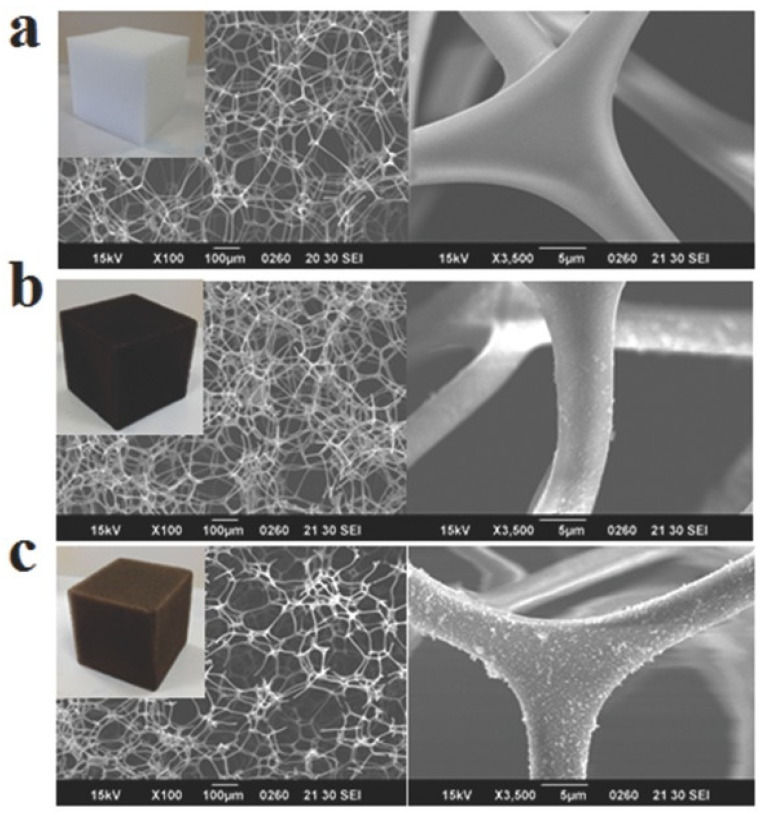
SEM Images and photos of (**a**) pristine MA, (**b**) PDA/MA treated foam and (**c**) Ag/PDA/MA foam [44]; reproduced with permission from John Wiley & Sons.

The less dense, porous, and widely available MA foams with high nitrogen content are hydrophilic and oleophilic in nature, thus demanding its modification for regulating the wetting properties. Though MA foams are less discussed in the literature than the PU, it is more appropriate to use as its porosity is higher (than PU foam) and possesses high absorption capacity [43]. Xu et.al. [44] were inspired by the hierarchical structure consisting of micropapillae of low surface energy, which contribute hydrophobicity. They selected dopamine to coat on MA surface, due to its strong and long-lasting adhesion to organic and inorganic surfaces. Figure 1 shows the SEM images illustrating the 3D porous structure of the original MA foam, unchanged by dopamine coating. However, the coating enhanced the surface roughness and the Ag nanoparticles (20 nm), resulted from metal binding ability of catechol in the polydopamine were uniformly distributed on the surface. The Ag nanoparticles and the PDA particles successfully build a hierarchical structure similar to the lotus leaf structure. The water CA of the as-formed Ag/PDA/MA was 160° and the water droplets rolled off easily from the foam surface. Whereas the PDA/MA without Ag, resulted in a contact angle values of 137.6° indicating its influence on wettability. Indeed, the absorption capacity of the foam was 60–130 times its weight even for high dense oils such as chloroform. The advantage of dopamine is its adhesion to different surfaces and its ability to provide a platform for secondary reaction that can connect to hydrophobic reagent to attain hydrophobic surfaces [45].

Various researchers utilized different functionalizing agents with dopamine to fabricate hydrophobic MA foams. For instance, Xiang et al. [46] used N-dodecylthiol (DT) as the hydrophobic agent due to its low surface energy in combination with PDA to coat MA. PDA nanoaggregates were formed through the self-polymerization of dopamine followed by covalent grafting of DT. PDA containing catechol structure can react with amino/thiol groups via Schiff-base reaction or Michael addition. The optimum PDA concentration was 8 mg/mL, and the modified MA foam exhibited a contact angle of 157° and a higher weight absorption of 5122 to 10,789 times its original weight. This treatment resulted in a 3D hierarchical structure loaded by submicron PDA nanoaggregates attached to DT with lower surface energy mimicking the topography of lotus leaf structure. Zhou et al. [47] reported higher hydrophobicity given by the contact angle for distilled water of 161° and absorption capacity of 6632–15,112% for 12 different oils by the same modification of DT/PDA/MA foam only by lowering the concentration of DA (3.0 mg/mL). The reason behind the superior results achieved by Zhou’s et al. is the high density of PDA nanoaggregates that at lower concentrations, PDA can be easily loaded in the foam. In contrast, the high PDA concentration resulted in the non-uniform distribution of aggregates. Though the thiol grafting to the PVA/MA foam resulted in superhydrophobicity, the whole grafting process was time consuming (12–20 h).

**Figure 2:**
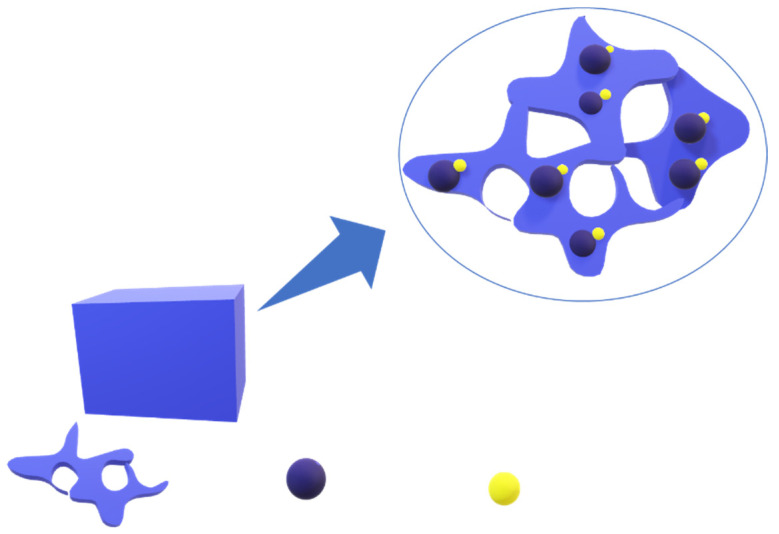
Schematic representation of MA foam modification by 1H, 1H, 2H,2H-perfluorodecanethiol and dopamine.

Ruan et al. [48] also explored the combination of PDA/MA with 1H, 1H, 2H, 2H-perfluorodecanethiol to attain superhydrophobicity. The water contact angle of the modified foam was superior (163.4°). It was proved that the sole coating material does not only contribute the water repellency as the coated platform had a contact angle of 97.3°. High porosity of MA (99.5%) increased the water repellent angle through the air trapped in its structure and the MA fibers retained quasispherical water droplets on its surface. The modified foam can absorb organic solvents and oils up to 97–195 times its original weight. Following the work of Ruan’s group, Shang et al. [49], decorated the PDA/MA foam by 1H, 1H, 2H,2H-perfluorodecanethiol molecules to generate hydrophobic foam, the process of which is schematically presented in Figure 2. However, during the coating process, after the immersion of MA foam in water/ethanol solution and prior the addition of dopamine, the foam was immersed in aqueous ammonia solution. The team emphasized the bridge relation between the amount of ammonia, hierarchical structure, and the resulted hydrophobicity. As the content of ammonia increases, the surface roughness increases according to and the size of PDA nodules, that contribute to the hierarchical structure. It is very important to keep the concentration of dopamine low (2 mg/mL in this research) as it yields high number of PDA nanoaggregates, but at the same time it makes the surface smoother and to counter this effect, ammonia in suitable concentration is utilized. Nonetheless, the as-modified F/PDA/MA foam (0.4 mL ammonia) showed water CA of 154° and absorption capacity of 68–172 times its own weight. The combination of PDA/MA with 1H, 1H, 2H,2H-perfluorodecanethiol molecules is very straight forward but the perfluoro-organic materials are toxic and is not highly recommended. In addition, the perfluoro-organic materials are indeed expensive which limit the utilization of such modified foams for large-scale applications.

Wang et al. [50] modified the PDA/MA foam using the grafting agent dodecyltrimethoxysilane (DTMS). With PDA/DTMS combined coating, the surface became rough while the sole coating of either PDA or DTMS made the surface smooth. The PDA caused for the nodules and the DTMS decreased the interfacial energy, with a high-water contact angle of 150° and the ability to absorb organic solvents 151 times the foam weight. 

Without the aid of adhesives, Chen et al. [26] modified the MA and PU foams of different densities by sole polydimethylsiloxane (PDMS) prepolymer due to its ability to irreversibly bind to the foams. The advantages of PDMS include its hydrophobicity, flexibility, and stability. Chen et al. also tried to test the effect of foam’s density on the oil/water mixture separation efficiency. For this purpose, MA foams with densities of 8 and 9.6 mg/cm^3^ and PU foams with various densities of 14, 19, 23 and 26 g/cm^3^ were utilized. Oil water separation efficiency monitored using vacuum pump set up became the highest when PDMS-MA (8 g/cm^3^) was employed as it absorbed silicone from silicone/water mixture and reached saturation within 15s. The water content in the collected oil was only 0.02%, indicating nearly 100% efficiency for the as-functionalized PDMS-MA. Conversely, the efficiency is much lower in the polyether sponge as some water droplets were presented in the soaked foam. The gap between the properties of the PDMS-MA and the PDMS-PU foams was also confirmed through the contact angle measurements. The PDMS-MA foams exhibited contact angle values of 150° for both densities, while it was 135° for the PDMS-polyether highest dense sample. The enhanced superhydrophobicity of the MA is attributed to the very high porosity of its structure (99.4%), higher than that of the polyether (97–98%) which had reduced the solid-liquid area contact and the adhesion area for the water droplets. Morphology analyses proved the pore size of MA and modified MA sponges in the range of 80−140 μm and 50−100 μm, and the density, 8 and 9.6 mg/cm^3^, respectively. Whilst the PU pore size was larger (210–970 μm) with larger ligaments (56–96 μm), finally proving that the smaller pore size and ligament yield higher porosity. Though the highest oil intake within 5s was attained by the PDMS-MA for all testing oils with 74 g/g for toluene, the absorption capacity reduces after the functionalization for both polyether and MA. This is because the thin PDMS coating increased, significantly, the weight of the foam, and the density of the PDMS coating film (965 mg/ cm^3^) is higher than the density of the raw foams (8–26 mg/cm^3^). 

On the other hand, limited number of researchers tested the functionalization of MA foam by nanoparticles (NPs). Basically, metallic, and carbonaceous NPs are anchored to the surface of the foam via mainly combination with organic materials or using microwave/ultrasonic deposition. It is worth knowing that without using these procedures, either the loss of the modifying layers or non-stable treatments are witnessed during the absorption/squeezing cycles or even poor absorption performance. For instance, Gao et al. [51] modified MA foam with silica NPs and vinyltrimethoxysilane coating (VTMS). While silica adsorption increases its surface roughness, VTMS imparts hydrophobicity. The dimension of the MA did not change after treatment and retained its 30–120 μm pore size. The absorption capacity of the modified foam was 60–109 g/g and the aqueous CA 150°. Superhydrophobic magnetic MA foam was obtained through coating its surface by Fe_2_O_3_ NPs via co-precipitation of FeCl_2_·4H_2_O and FeCl_3_·6H_2_O using ammonia catalyst in the presence of stearic acid [52]. The presence of stearic acid in the reaction led to attachment of -(CH_2_)_16_CH_3_ into the Iron (III) oxide NPs and this high carbon content media significantly reduced the surface energy of the foam. Fe_2_O_3_ NPs made the foam’s surface rough and hydrophobic (>150° WCA) and enhanced its superparamagnetic property. The foam functions as oil sorbent with high efficiency through manipulating it on the oil regions in oily water by a magnetic bar. The sorption capacity of the magnetic Fe_2_O_3_/MA was 17–80 g/g, but non-magnetized modified sponges also possessed high sorption capacity [48]. This indicates that magnetization can be critical in terms of oil sorption efficiency, for instance, manipulating the magnetic foam via magnetic bar enabled the foam to absorb hexane from viscous water/hexane mixture (0.02:1 *v/v*, viscosity of 0.22 Pa S) within 2 s. However, Song et al. [53] reported remarkable results such as absorption capacity of 80 g/g and WCA of 154° by treating MA foam with reduced graphene oxide (rGO) under ultrasonic and microwave radiation. The rGO also increased the surface roughness. Though the NPs modification of MA perform quite high WCAs and absorption capacities (75–90 g/g), the results are not significantly different from the modification by organic compounds, except the poor reusability of NP modified foams.

**Figure 3:**
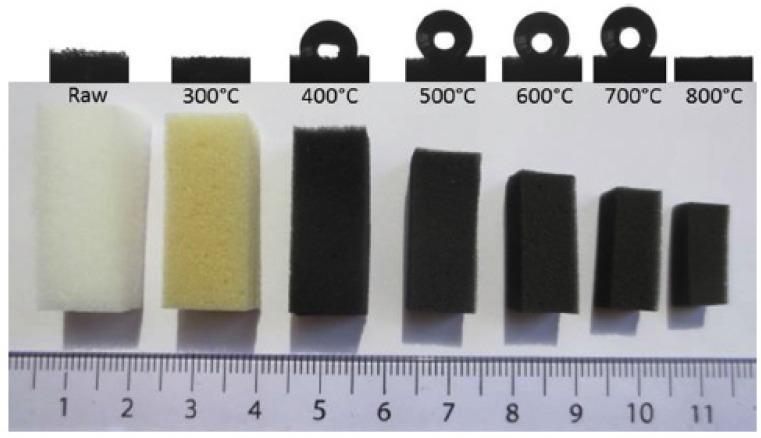
Photographs of neat and carbonized (300–800 °C) MA foams along with aqueous contact angle images. At 300 °C and 800 °C, water droplets rapidly absorbed by the foam [54]; reproduced with permission from ELSEIVER.

Carbonization is another technique for fabricating hydrophobic sponges, as reported by Stolz et al. [54]. Best carbonization temperature was identified as 500 °C–600 °C for 1 h at the rate of 10 °C/min. Pyrolysis reduced the apparent density of the foams from 8.3 mg/cm^3^ to 6.7 mg/cm^3^ and the pore size by a factor of 1.7 and 2.9 for 400 °C and 800 °C respectively. The thermal degradation behavior of the foam was identified as (i) ˂350 °C water evaporation, elimination of formaldehyde from ether and methylene bridges formation, degradation of unbonded MA etc. (ii) at 350–400 °C methylene bridges break and enhance dimerization reactions with emission of nitrogen gas (iii) at 400 °C up to 600 °C MA inter-ring condensation and a rejection of ammonia and (iv) at 600 °C, recombination of triazine rings. The carbonized foam retained the triangular fibrous structure with~ 99.5% porosity. But elevated temperatures affected the fiber dimension and reduced the porosity and pore size. Since carbonization did not cause any roughening in the structure, the hydrophobic wettability of foams at 500–600 °C is attributed mainly to the reduction of surface energy by the formation of disordered hydrophobic graphene like aromatic carbon planes arrangements. The optimum wetting response is demonstrated in the Figure 3 at various temperatures and highest value of 120°–140° is achieved at 500 °C. At 400 °C and 700 °C, the foam was neither fully hydrophobic nor hydrophilic while at 800 °C and 300 °C, it was hydrophilic (at 300 °C the foam did not undergo carbonization and at 700–800 °C the foam experienced deposition of Na_2_CO_3_ molecules which altered its surface energy). Most importantly, the foam carbonized at showed high oil capacitive performance of 90–200 time its own weight towards different organic solvents.

Chen et al. [55] investigated carbonization of MA foams and its effect on surface wettability Pristine MA foam (untreated and without any additives) was carbonized at 700°, 800°, 900°, 1000° and 1800 °C for 0.5 h at 5 °C/min. The sponge carbonized at 800 °C was the most elastic and highest capacitive among the rest and its superhydrophobicity allowed it to float over water while separating diesel oil from its watery mixture. This observation is opposite to the findings of Stolz et al., who reported that the 800 °C-carbonized sponge was superhydrophilic. However, both performed a treatment on the neat foam, with only difference in the pyrolysis duration (1 h and 0.5 h as per Chen and Stolz procedure). Nonetheless, at temperature of 800°, the foam showed excellent absorption capacity of 148–411 times its original weight towards different organic solvents and high porosity (99.6%). Indeed, the carbonization process greatly reduced the foam density from 7 mg/cm^3^ into 5 mg/cm^3^ for the 1800° carbonized MA due to the low carbon yield (~9%).

**Figure 4:**
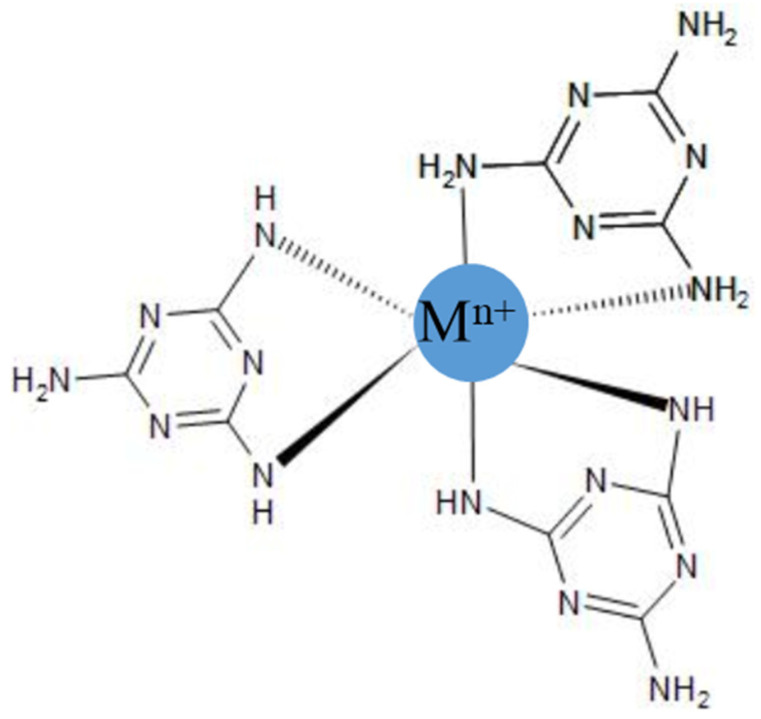
Coordination interactions between metal ions and N atoms in melamine foam structure.

Ding et al. [56] introduced a facile fabrication method for the porous hydrophobic MA (ρ = 10.16 mg/cm^3^) through short time immersing of intrinsic foams in various transition metal ion solutions such as FeCl_3_, Fe(NO_3_)_3_, Ni(NO_3_)_2_, Zn(NO_3_)_2_, and Co(NO_3_)_2._ The coordination of transition metal within the MA structure induces hydrophilic to hydrophobic transition. Since MA foam is made of repeating 2,4,5-triamino-s-triazine structural units, it is rich in nitrogen atom content that have unpaired electrons in its structure. These lone-pair electrons enhance the polarity of the foam, and thus the hydrophilic wettability. Transition metal ions form coordination complexes through covalent bonding and reduce this polarity along with surface energy (Figure 4). The ability of the transition metals to switch the wettability of the material is baffling as very low concentration can produce superhydrophobic wettability. For example, 0.05 mM FeCl_3_ treated foam has superhydrophobic surface with water repellent angle of 130° ± 5.1°. Reasonable concentrations of FeCl_3_ (0.005 M up to 0.1 M) did not change the morphology of the foam and it retained its smooth open cell structure while for excessive concentrations of FeCl_3_, roughness in some points on the surface was observed. However, the metal ion induced foam shows oil absorption capacity of 71–157 times its own weight.

**Figure 5:**
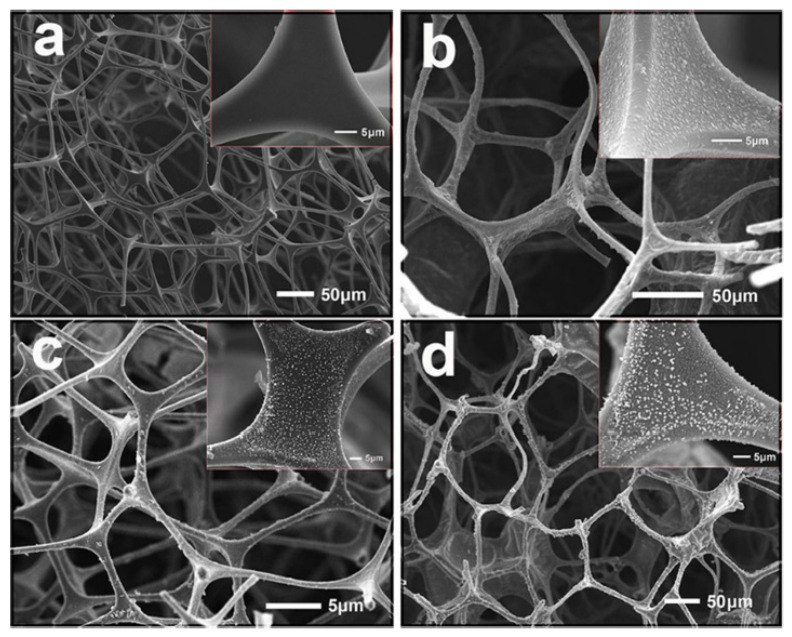
SEM images for (**a**) pristine MA foam, (**b**) PPy/MA, (**c**) PPy/Ag NPs/MA and (**d**) PPy/Ag/fluorinated MA [57]; reproduced with permission from ELSEVIER.

Chen and his coworkers [57] prepared two tier rough MA foam through three different modification methods involving immersion in ethanol/FeCl_3_ oxidant solution, vapor phase deposition of pyrrole on the foam surface and treating the Fecl_3_/PPy MA2 foam with AgNO_3_ solution. This results in the reduction of Ag NPs onto the surface constructing the second tier of roughness and finally the foam was immersed in fluoroheptyl-propyl-trimethoxysilane solution. Indeed, the formation of PPy particles (0.2–0.3 μm) and Ag NPs (50–80 nm) on the surface of the foam served in constructing the two tiers of roughness as shown in the Figure 5. While the PPy deposition generated hydrophobic foam with contact angle of 126.6° Ag NPs decreases the value to 112.3°. The final fluorination process again significantly increases the hydrophobic angle up to 156°. Although the presence of Ag NPs slightly reduced the WCA, as per Chen team’s control experiment the contact angle for free Ag NPs PPy/Fluorinated MA was much lower than that of the PPy/Ag/fluorinated foam (139.5°). However, the absorption capacity of the as-formed foam ranged 39–100 for 12 different oils.

Superhydrophobic MA sponge for an efficient absorption of oil spills was described in [US20170036190A1] by Viet Hung Pham, James Henry Dickerson [58]. That disclosure involves the preparation of hydrophobic and super-hydrophobic MA sponges having a good mechanical strength, superior recyclability, manufacture scalability, and low cost. The hydrophobic or super-hydrophobic mixtures consist of alkylsilane ora fluoroa kylsilane having an alkyl group of different length. The preparation of MA sponge suitable for oil in water separation was proposed in the invention of YU-HSIANG LIU and SHIH-CHUNG CHEN [59] [US16039970]. The foams were synthesized was prepared using polyol blend with a polyisocyanate and graphene, with an addition of a catalyst, a foaming agent and a foam stabilizer.

### 2.2. PU Based Foams

**Figure 6:**
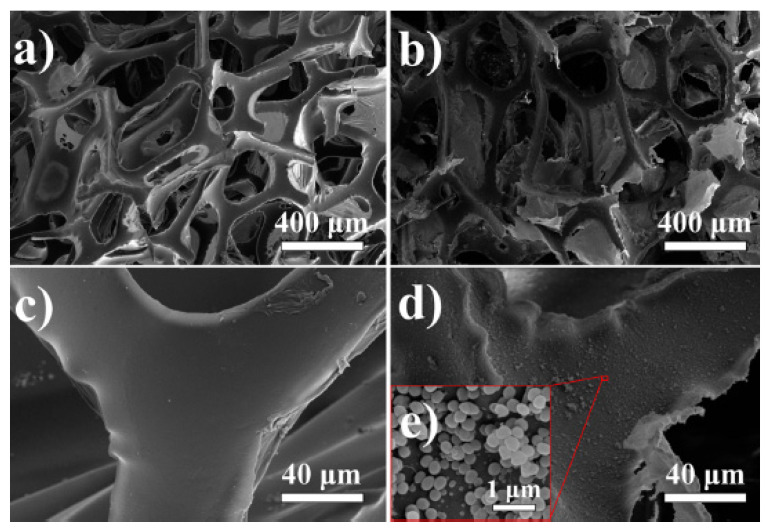
(**a**,**b**) typical and magnified SEM images for the neat PU and modified PU foam; (**c**–**e**) with DT and DA [61]; reproduced with permission from the American Chemical Society.

PU foams are commercially available strong materials suitable for oil/water separation applications after appropriate modifications. PU foams are low in cost, have excellent mechanical properties, low density, large pore volume and wear resistance. Since the wettability is controlled by the surface roughness and structure, it is necessary to modify PU foams by regulating its surface roughness and structure. However, the modification of PU foam is very similar to that of the MA foam; but the results are not the same for the two modified foams. In this section of the review, different modifications of PU foam are discussed, and the efficiencies are compared with that of MA foam [60]. Taking the advantage of dopamine deposition by polymerization of all substrates, Li et al. [61] fabricated superhydrophobic durable foam through the modification of PU with dopamine, Ag NPs and dodecylmercaptan (DM). In the modified foam, the dopamine level was very high, Ag NPs were modified through the reduction of n-butylamine, and further modification was done in solution containing DM to decrease the surface tension. Among the three modifications, DM/Ag/PU foam resulted in the highest hydrophobicity (155° CA) and the adsorption capacities ranged from 18–43 g/g. Though the authors did not mention about the porosity of the foam, observed morphology images prove significant surface roughening and surface tension decrease and thus those two elements are regarded as the source of superhydrophobicity. More recently, Huang et al. [61] modified PU foam with PDA and n-dodecylthiol without affecting the pore size (200 to 340 μm), but the surface became rougher (Figure 6). The contact angle was 157° and its weight gain was 2494% to 8670% for 12 different oil/organic solvents. The modification was done with the same DA concentration (8 mg/mL) similar to the Xiang’s team [46] on MA as discussed in Section 2.1. In both cases, the contact angles reached were the same, and the weight gain was outstanding for the DT/DA/MA foam (5122–10,789%). Though the modifying materials and the DA concentration remained the same, the superior performance of MA foam can be attributed to its high porosity. 

**Figure 7:**
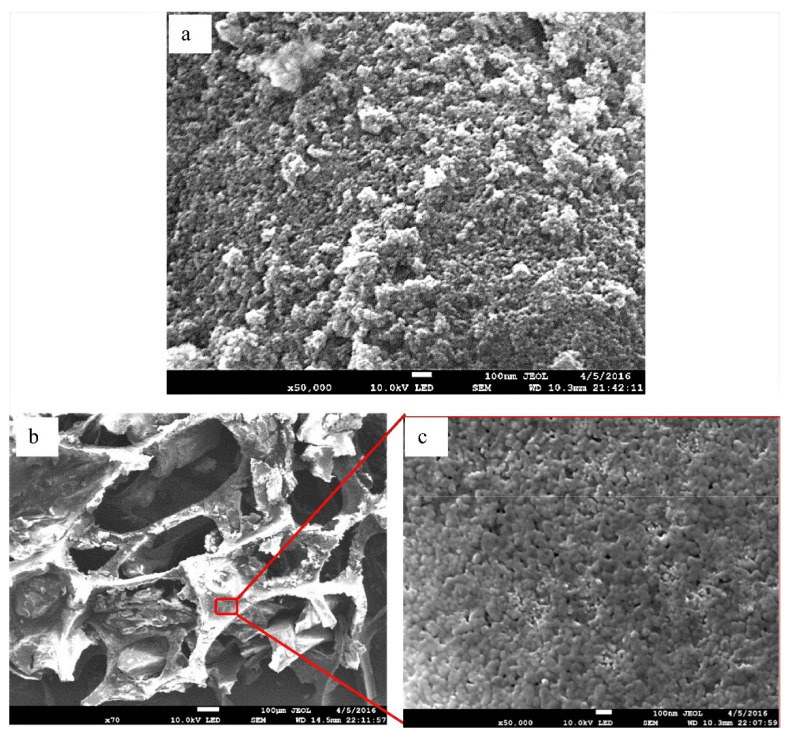
FE-SEM Images of the (**a**) OH-NDs-fPDA powder; (**b**) coated OH-NDs-fPDA-PU foam and (**c**) zoomed in OH-NDs-fPDA particles on PU foam [62]; reproduced with permission from ELSEIVER.

Cao et al. [62] modified PU with hydroxyl terminated nanodiamonds (34 nm), 1H,1H,2H,2H-perfluorodecanethiol (PFDT) and PDA. The loading percentages of OH-NDs-fPDA on PU foam was found to be very critical as increasing the loading up to 60%, increased the contact angle up to 150°. The topography of the foam found to be very rough with highly agglomerated OH-NDs-fPDA and irregular pores (Figure 7). The absorption capacity was also 15–60 times its own weight. However, without the utilization of OH-NDs, Ruan and Shang achieved very good results of 154° WCA and 68–172 higher absorption capacity than its own weight by PFDT/PDA coating on MA. 

**Figure 8:**
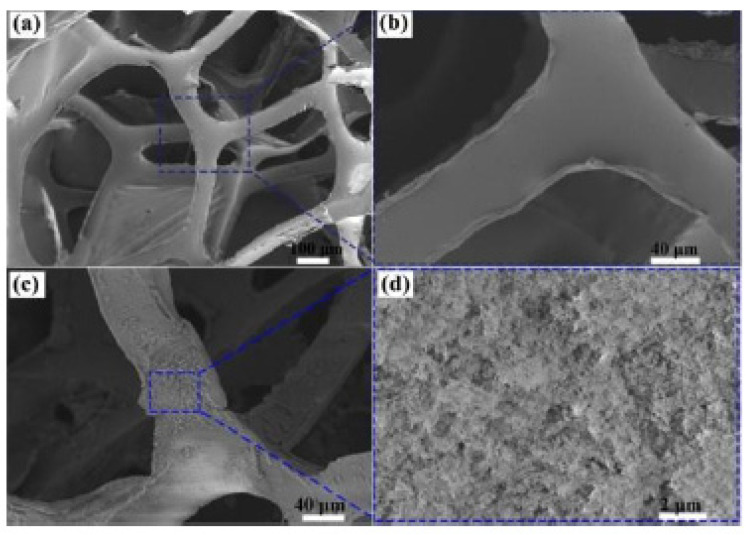
Low (**a**) and high magnification (**b**) SEM images of pristine PU foam; Low (**c**) and high magnification (**d**) SEM images of SiO_2_/VTMS PU foam [64]; reproduced with permission from the American Chemical Society.

Modification of PU foam via salinization process is less discussed in the literature. Nonetheless, Xiong et al. [63] coated the PU foam by alkylsilane coupling agent. The alkylsilane agent couple with the PU foam through the isocyanate group existing on the surface of the foam. The WCA and the absorption capacity of the alkylsilane immersed PU sponge were obtained as 137° (hydrophobic) and 75 times its own weight, however, the achieved results were lower than those obtained for alkylsilane coated MA by Pham and Dickerson [58]. Indeed, the alkysilane coated MA is superhydrophobic with contact angle 151° and has an absorption capacity of 82–163 greater than its own weight. The treatment of PU foams by nanomaterials to attain superhydrophobicity was done via different methods. Li and his team [64] coated PU foam with SiO_2_ nanoparticles physically to prepare a hydrophobic foam that absorb oil up to 55.8 its own weight with a contact angle of 130°. The topography of the modified foam showed a porous structure of 100–400 μm, the foam’s fibers become rougher and close packed with silica NPs as shown in Figure 8. However, the SiO_2_/VTMS treated MA foam shows higher efficiency than PU/silica NPs. This could be attributed to the smaller pore size of MA sponge (30–120 μm) which could prevent water drops from entering the skeleton of the foam. 

Reports also show superhydrophobic PU foam loaded by carbonaceous NPs by ultrasonication treatment [65], exhibiting a water contact angle of 127° and absorption capacity of 121 times the original weight of the foam. Moreover, rather than the simple addition of NPs to enhance the roughness of foam’s surface, magnetic properties were also induced by some additives. It is believed that the added magnetic properties can create excellent contactless oil clean up. Anju and Renuka [66] prepared graphene meso Fe_3_O_4_ composite incorporated PU foam. The foam, after treatment, exhibited crumpled sheets of graphene loaded with Fe_3_O_4_ particles, which benefit enhancing the roughness of the foam. The presence of graphene sheets is crucial to stabilize the modified foam by retaining the iron oxide particles cohesive to the foam. The presence of mesoporous iron oxide particles not only help in roughening the surface, but it also stores the oils in its mesopores, which create extra volume for the oil storage. The paramagnetic behavior of iron oxide particles further facilitates the oil sorption by electrostatically attracting the polar compounds in the oils and other organic solvents. The water droplets exhibited quasi-spherical shape on the modified foam with a contact angle of 151° and its adsorption capacity ranged between 90–310 g/g for different oils.

Cement coating was applied on PU, to fabricate superhydrophobic sponges by Zhang et al. [67] and achieved a water contact angle of 155°. The PU was simply immersed in hydraulic cement/stearic acid/ ethanol solution followed by heating at 50 °C for 1 h, with an optimum cement: water ratio of 0.5. Cement coating improved the oil absorption by providing rough surface and lowering the surface energy via assisted STA modification. The absorption capacity of the as-fabricated foam was extremely high as the fabricated foam capacity became 2500–3500 times its own weight for four different organic solvents (hexane, dichloromethane, peanut oil, and diesel oil). However, it can be concluded that either by using the same sole modifying material for PU and MA or by using same modifying material but in a different composite outstanding performance is always achieved. It is believed that the main contributor to the difference in performance is the quite high porosity of MA foam (99.4%) compared to PU (97–98%) [26].

Z.J. Kozlowski [68], in US5,239,040 described a separation of oil components from emulsified water to meet potable water standards using PU foams in the form of microspheres. PU foams have been modified by various reactants using specific processes and effectively insured very low level of non-aqueous phase in the effluent. The absorbent has been found suitable for use in cleaning up spilled liquids including gasoline, and crude oil. This concept was employed for a development of polyurethane oil de-emulsification unit (A. Benachenou, J.P. Parent, US8,721,895 B2) [69].The incorporation of PU foams into a percolating biological filters for oil-water separation was described by C.A. DE LEMOS CHERNICHARO and P.G. SERTÓRIO DE ALMEIDA in the invention [70][WO2014063219A1].

## 3. Oil/Water Emulsion Separation by MA and PU Foams

Appropriate modification of MA and PU foams is very crucial to turn their wettability towards oil/water emulsion separation applications. The modified foams are expected to selectively absorb oil from a typical emulsion and repel the water and vice versa. In this section, the utilization of modified MA and PU foam for separating both oil in water emulsions and water in oil emulsions is discussed. 

### 3.1. Separating Oil in Water Emulsions

**Figure 9:**
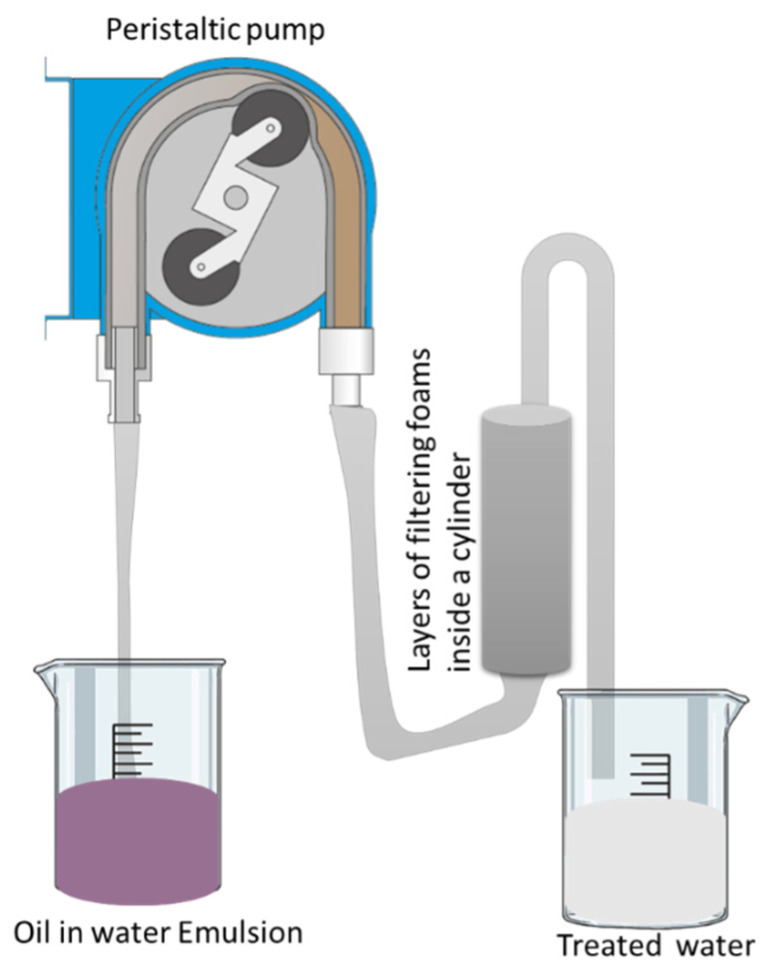
Scheme of the separation unit and filtrate of the oil in water emulsion.

Continuous discharge of oily wastewater into major water bodies is a serious harm to human health [71,72]. This demands the efficient treatment of oily wastewater prior to releasing it in the environment and for that purpose; strict standards for the oily wastewater discharge are regulated by many countries. Generally, the oil present in wastewater can be: (i) floating oil with the largest droplets among the other types (≥150 μm), (ii) dispersed oil (20–150 μm), (iii) emulsified oil (˂20 μm) and (iv) the dissolved oil or ‘’water-soluble oils’’ such as organic acids and phenol derivatives [73,74]. However, emulsified oil is the most harmful and difficult type to separate. The main principle behind the filtration materials, which are used to withdraw oil from water in the emulsions, is the pore diameter of the material. To this end, there are different available techniques for the separation process including, textiles [75] and metallic meshes [76,77]. Nonetheless, the aforementioned techniques suffer from the high cost of fabrication besides the fouling up impediment. The commercially available porous 3D polymeric foams satisfy the need for low cost, and recyclable filter material. In this regard, many papers have discussed the modification of available MA and PU foams for the separation of oil in water emulsions (OWE) including surfactant stabilized emulsion and the free-surfactant type. For instance, Kong et al. [78]. prepared hydrophobic PU foam through in situ polymerization of graphene in the presence of N-methylpyrrolidone (NMP). The foam exhibited 99.9%, 99.96% and 99.91% respective efficiencies for Tween 80 surfactant-stabilized hexane, hexadecane, and soybean in water emulsions. Very recently, Han et al. [74] coated MA with graphene/PDMS composite to prepare a superhydrophobic filtration material. Han’s team used bottom-to-top approach to pass the emulsion through the graphene/PDMS MA foam as shown in Figure 9. For the treated water of 25.6 mg/l oil content and 4% (volume ratio) initial oil concentration in the emulsion, the oil removal rate remained higher at 99%. When the foam thickness is increased, the efficiency gradually increased, however the optimum thickness was 5.4 cm among the other tested thicknesses (1.8 and 3.6 cm).

Robust hydrophobic PU foam was obtained by coating with attapulgite (APT) as illustrated by Li and his colleagues [79]. The foam exhibited excellent efficiency up to 99.87% for the separation performance towards five kinds of Tween 80 stabilized oil in water emulsions. Indeed, the oil droplets of the filtrate were of less than 78 ppm dimension except for the diesel/water emulsion (112 ppm). In another report, superior results in the emulsion separation were achieved by Khosravi and Azizian [16] via coating PU foam with polypyrrole (PPy) and palmitic acid (PA) solution. This coating slightly decreased the porosity of the foam (90 to 88%), but it increased the pore size (0.5 to 1 mm) and the pore volume (18.6 to 18.9% cm^3^/g). The foam was demulsified by dipping in toluene/water emulsion for 2 min (efficiency was 100%), and further tested for oil cleanup using crude oil/water mixture (within 2 min the environment was cleared out of oil). In such systems, the driving force for the demulsification is the intermolecular forces “London force” between the hydrophobic foam and the non-polar oil droplet in the emulsion [80]. In addition, Zhang et al. [80] modified the MA foam by immersing it in ethanol, tannic acid and n-octadecylamine solution, and it was used to separate both toluene and n-hexane in water emulsions. The filtrate was clear without any oil droplets and the efficiency of the foam was 99.57% based on the oil rejection rate (R%) according to Equation (7):

(7) R (%)=(1 − C_p_/C_o_) × 100%

**Figure 10:**
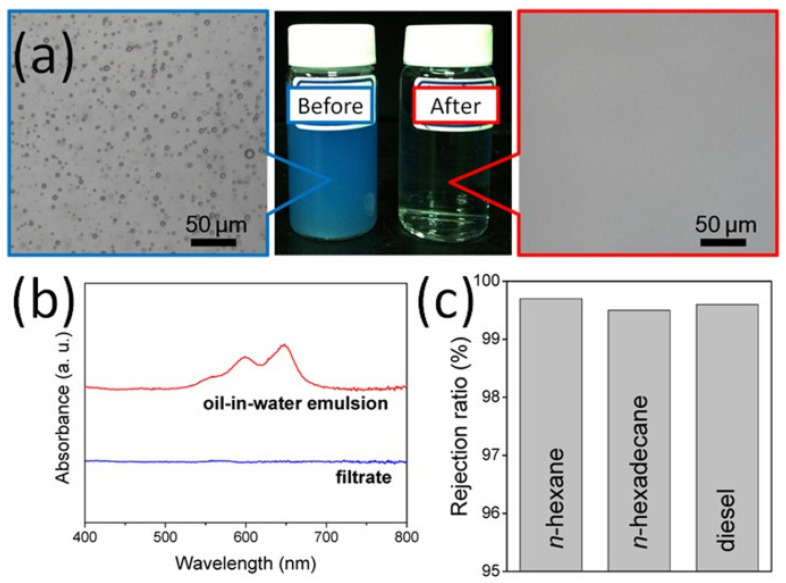
(**a**) Photographs and (**b**) UV−V are spectra of the surfactant-free n-hexane–in–water emulsion before and after separation using compressed protonated MA sponge; (**c**) Oil rejection ratios of three kinds of filtrates collected from their oil-in-water emulsions [81]; reproduced with permission from Springer.

Figure 10 represents the separation efficiency of both surfactant-free and -stabilized (Sudan Blue II dyed) oil-in-water emulsions by protonated MA sponge [81]. Interesting fact is that the separation process was continuous up to 12 h without changing the superhydrophilic, underwater superoleophobic and antifouling properties. Though the pore size of MA sponge was ˃50 µm, it was not effective in separating the emulsions due to the small droplet size of ˂20 µm. Therefore, compressing technique was applied on the MA sponges so that the pore size was reduced and the MA skeleton became compactly packed surfactant stabilized emulsion. The separation performance was excellent as clear by the absence of any droplets in the filtrate, and also the oil rejection ratio was observed to be ˃99.5% with no characteristic peaks of the dye in the UV-vis spectrum. 

**Figure 11:**
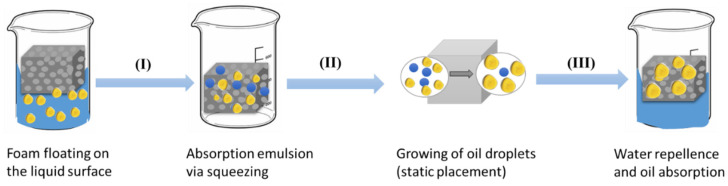
The mechanism of oil in water emulsion separation by modified PU/MA foams.

Wang et al. [82] decorated PU foam with dodecanethiol (DT), fly ash (FA) and dopamine to test its efficiency of separation to six kinds of surfactant free oil in water emulsions. The whole process was done by simple immersion of the foam into the prepared emulsions and an efficiency of 93% was achieved. The contact angle and the absorption capacity of the fabricated foam were also very high (161°, 34–47 g/g) and the fabricated foam has the advantages of flame retardation and oil/water separation efficiency in corrosive media. However, the performance was poor when compared with other reported modified PU foams. The mechanism of separation process was explained according to the Figure 11, by which the foam floats initially on the surface of the emulsion due to its hydrophobicity (Figure 11I). However, by squeezing in the foam, the emulsion was sucked into the foam skeleton through its pores, and it causes instability in the emulsion droplets (Figure 11II). Thus, the oleophilic foam continuously absorbs the oil droplets via intermolecular interactions and forms colloids by merging the oil droplets(Figure 11III), while the water is repelled out of the foam skeleton due to hydrophobicity of the foam and clean water filtrate will be resulted by the end of the sorption experiment (Figure 11III) [80].

### 3.2. A Separation of Water in Oil Emulsions

**Figure 12:**
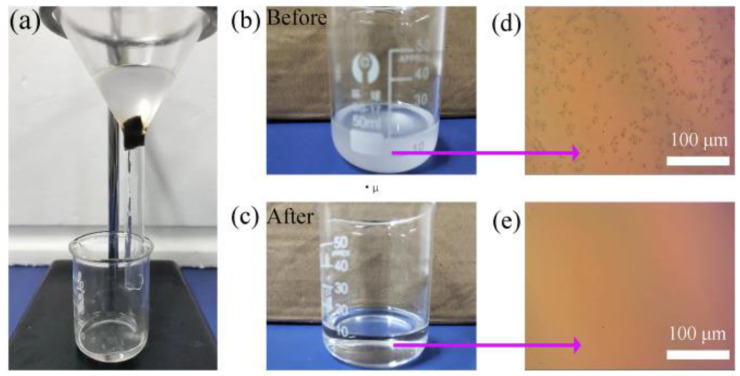
Photographs and optical microscopy images of a surfactant-stabilized water-in- chloroform emulsion before and after separation [83]; reproduced with permission from ELSEIVER.

Oil in water emulsions consist of oil droplets surrounded by water phase, whereas the water in oil emulsion has water droplets captured by oil. Water in oil emulsions is prepared by mixing small amounts of water in comparatively larger amounts of oil medium and Zhou et al. tested the efficiency of silk fibroin-graphene oxide functionalized MA sponges in separating such an emulsion stabilized by a surfactant [83]. Here, the silk fibroin act as a molecular binder in developing the hydrophobic sponge, and the functionalized sponge separates emulsion by gravity driven method (Figure 12a). Through the sponge, the oil phase easily penetrates while the water phase remains. While the original emulsion remains milky white (Figure 12b), the filtrate is clear (Figure 12c) with a separation efficiency of 93%. The optical microscopy images further evidence the clarity of separation, as there are no water droplets observed in the filtrate (Figure 12e) compared to the optical image of the emulsion (Figure 12d). In addition to the emulsion separation, the composite exhibited superior oil adsorption capacity (up to 76 times its own weight), mechanical properties (100 cycles), and recyclability (50 times).

The most recently developing MXene 2D material was combined with tetradecylamine (TDA) by Xue et al. [84] to develop superhydrophobic foam. Separation of the emulsion was done by keeping the foam in a syringe and compressing to release the filtrate. The efficiency of the process was estimated to be 96.88% for Span 80 stabilized water in toluene and dichloromethane emulsions and the flux reached up to 1500 L/m^2^.h and 1800 L/m^2^.h respectively. This outstanding performance was also correlated with the high surface area of MXene. However, superior results were achieved via superhydrophobic and magnetic PU sponges by Guselnikova and his coworkers [85]. They modified the PU foam with 3,5-bis(trifluoromethyl)benzenediazonium tosylate and diazonium salt particles and tested against a series of surfactant -stabilized (Pluronic F-127 surfactant) and -free emulsions. The immersion time in the water in oil emulsions was varied from 5–8 min depending on the emulsion type. No oil droplets were observed in the water, which indicated a total efficiency of 99%.

**Figure 13:**
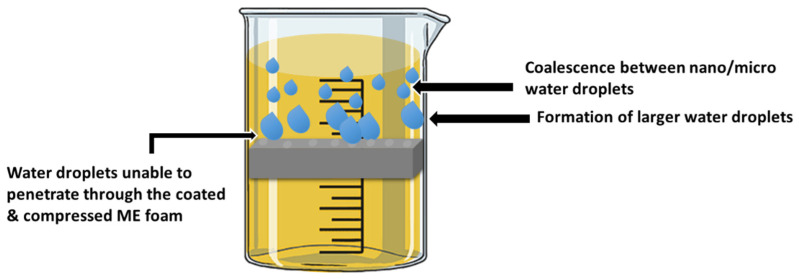
Schematic representation of water in oil emulsions separation by modified PU and MA foams.

Yang’s team modified the wettability of MA foam via coating by polyethyleneimine (BPEI) and dipentaerythritol pentaacrylate (5Acl) [86]. They observed that the 1,4-conjugate addition reaction between amine and acrylate groups boosted the surface roughness and the reaction between the nano-complex solution and octadecylamine reduced the surface energy. Furthermore, the coated foams were compressed mechanically at different degrees (0%, 20%, 40%, 60%, 80% and 90%), to reduce the pore size and to meet the demulsification requirement. The compression technique practically increased the separation efficiency of the modified foam by 9.14% at 90% compression. In other words, the efficiency of the sole coated MA foam was 90% for clearing surfactant-stabilized water in hexane emulsion while it was 99.14% in the coated & compressed MA foam for the same emulsion. The major findings from this research were that (i) water droplets size and foam pore size are key factors for the demulsification process and (ii) compression decreases the pore size helping the modified foam to capture oil, spread it out via capillary forces and to reject water droplets and interrupt it. However, at ˃90% compression, both modified and compressed foam were able to separate effectively due to calescence. When the demulsification process takes place, the water droplets in the original emulsion coalesced with each other to foam larger drops, which will be rejected by the smaller sized pores of the compressed foam (Figure 13). On the contrary, in the absence of compression step and by sole coating process, the coalescence of water droplets would not occur due to the stability of the emulsion, which devalue the separation performance. 

**Figure 14:**
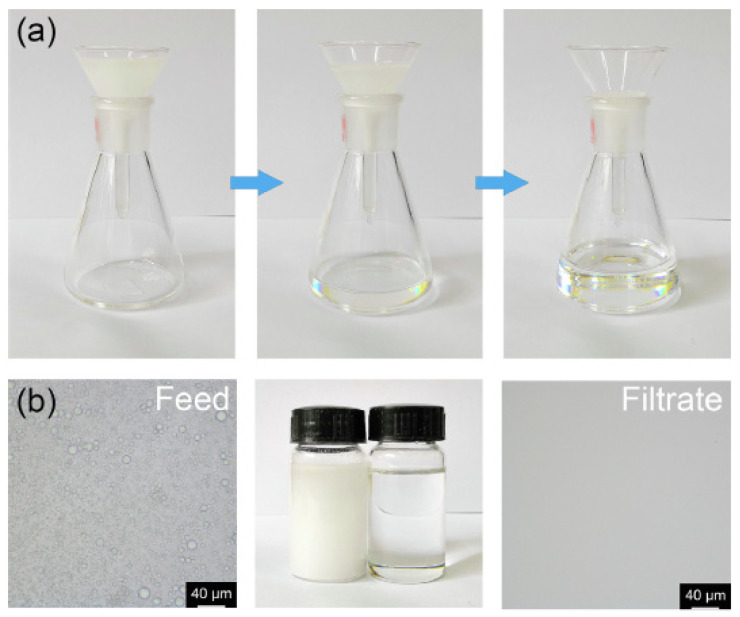
The separation process of water in toluene emulsions (**a**) photographs and (**b**) optical microscopy images of the feed water and filtrate [87]; reproduced with permission from ELSEIVER.

Acrylate building blocks were also used by Li et al. [87] for coating the MA foam by acetylic/ silica copolymer. Five different Span 80 water in oil emulsions were prepared (toluene (560 nm water droplet size), gasoline (421 nm), chloroform (385 nm), diesel (577 nm) and n-hexane (622 nm)) and the simple gravity demulsification process of the as-prepared foam exhibited a total separation efficiency of 98% (Figure 14).

In the same way, carbon nanotubes and PDMS coating were used to switch the wetting properties of PU foam by Wang and his coworkers [88]. Vacuum filtration was applied to separate water in n-hexane, n-hexadecane, and gasoline emulsions and respective efficiencies of 99.99, 99.99, and 99.97% were achieved. In addition to all the explained research, an excessive number of papers reported the usage of different modification methods for MA and PU foams in oil/water mixtures separation. Though few numbers address the challenges of the modified foams, a table is provided to summarize all recent studies (Table 1). 

### 3.3. Recyclability and Durability

Modification of commercially available polymeric foams to completely target their oil/water separation ability, is majorly related to the cost of production/processes, durability (chemical stability) and mechanical robustness. In general, the durability of any foam is evaluated by the cyclic abrasion test which include placing the foam in harsh environments or sometimes in different temperatures for a certain time followed by measurement of the water contact angle of the foam. Indeed, repeated compression test are employed to further test the mechanical robustness of the foam. Recyclability is determined by the ability of the foam to keep its performance of oil sorption after numerous absorption/squeezing cycles. If the foam is still in contact and able to separate oil/water mixtures after great numbers of absorption/ squeezing cycles, the foam is described to be reusable/ recyclable. However, the deuteriation resulted from abruption/squeezing cycles test is gradual and most of the reported foams in the literature witnessed weakening by 10% after the highest recorded cycle’s number.

**Figure 15:**
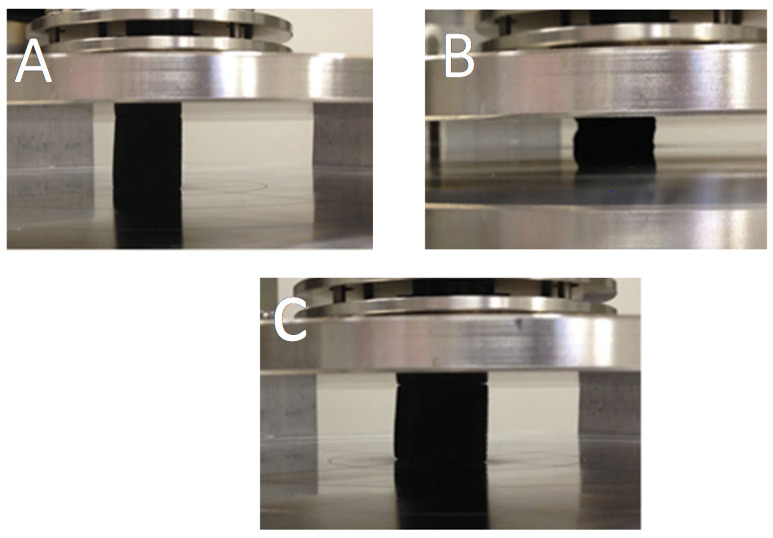
Sequential images of compression at 30%, 60% and 50% for the fluoroalkanethiol/dopamine coated MA foam [44]; reproduced with permission from ELSEIVER.

Robust superhydrophobic MA foam was produced through modification via fluoroalkanethiol and dopamine [44]. The robust foam was able to recover from compression strains of 30%, 50% and 50% as shown, respectively in Figure 15A–C. Indeed, the fabricated foam was tested by absorption/squeezing using n-hexane in water mixture and it remained unchanged even after 10 cycles and retained the hydrophobic angle of 153° beyond cycles 10. Furthermore, the modified foam underwent abrasion test 100 times by sandpaper, and it witnessed little deformation beyond 100 with the resulting contact angle of 146°. Thus, such foams satisfy the recyclability and durability parameters, which introduces the foam as a strong candidate for real oil spill cleanup processes.

**Figure 16:**
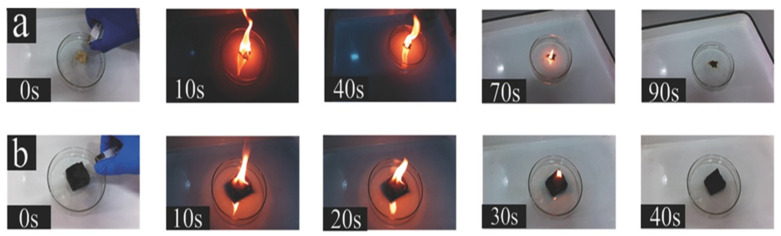
Video snapshots of the combustion of (**a**) MA and (**b**) rGO/MA containing adsorbed gasoline [107]; reproduced with permission from ELSIVER.

Indeed, even higher robustness was achieved for the rGO/MA hydrophobic foam by Zhau et al. [107]. Its absorption capacity was140 times its own weight, and the mechanical stability was outstanding as it recovered to its original shape after 100 cycles at 50% compression. Chemical stability was confirmed by keeping the foam in different oils and organic solvents for 12 h, while the thermally stability was tested by igniting the watch-glass slice containing adsorbed gasoline with the foam. When MA and PU are compared for thermal stability, rapid ignition was noticed in the pristine PU foam (90 s to be fully burned) whereas the rGO/MA foam build up the fire on its surface gradually and by 40 s it was completely burned off (Figure 16). Since most of the oils and organic solvents are flammable liquids, flame- retardation ability becomes great important to avoid any expulsion or fire that can be caused by improper handling. However, the combustion retardation resistance of the MA foam is because of its ability to release inert gas during combustion unlike the PU, which burn itself. The recyclability of the rGO/MA foam was also tested by manual absorption/squeezing method using toluene, and for 50 cycles no deterioration was noticed. However, this was not the same with pump oil, as sharp decrease in the absorption performance after the second cycle was noticed due to oil trapping in the foam skeleton.

Wang and Deng [106] explored the recyclability and durability of carbonized hydrophilic MA foam. Although, robustness of such sponges was questionable, the 1H, 1H, 2H, 2H-perfluorodecanethiol coating on carbonized foam possessed great chemical stability as evident from the high-water repellent angle ˃ 150° even after immersing it in various pH solutions (pH 1, pH 7 and pH 13) for a day. Environmental stability was tested by placing the fabricated foam in various harsh environments including liquid nitrogen (−196 °C), and air (200 °C) for 2h and UV irradiation (λ = 365 nm, 500 W, 24 h). Even after the harsh environment treatment, the foam only showed a slight decrease in its contact angle compared to the initial value (156.8°). Moreover, the foam retained high WCA of 154.6° after 50 cycles of abrasion. Tetrachloromethane and n-hexane were used for absorption/squeezing tests of the foam for 15 cycles and no obvious damage was noticed on the foam while slight decrease in the WCA was noticed (158.5°–154.5°). Though the authors checked the practical chemical and environmental resistance of the as-prepared foams, the mechanical stability (compression) tests were not addressed. 

Decoration with cellulose nanocrystals (CNCs) is done as an additional step, after carbonization by Lei et al. [108]. The carbonization conditions were fixed as 1 h pyrolysis under nitrogen atmosphere at 400 °C. The CNCs carbonized MA foam showed outstanding recovery beyond 100 cycles, by retaining 97.4% of its original oil sorption capacity with high contact angle (151.1°). These results were higher than those reported by Wang and Deng [82]. The foam also showed great thermal stability as it did not lose weight after burning under air at 450 °C, and it did not ignite when subjected to alcohol lamp flame. This was attributed to the high nitrogen content in the foam, which enabled it to release air penetration molecules as NO, NO_2_ and NH_3_. Indeed, the foam was subjected to 100 cycles of mixed oils absorption/combustion, and it still maintained its structure and 91.9% of its absorption capacity. Therefore, the introduction of CNCs into the carbonized foam was found to be of particular importance for developing its performance efficiency. In addition, the stress vs strain curves for compression at 60% over 1000 cycles were monitored for the modified foam and higher stress by 4.5 KPa compared to the sole carbonized MA was noticed. The CNCs/carbonized MA foam retained 98.9% of its original stress value over 1000 cycles of compression at 60%. 

Based on the above discussions [106,109], it is clear that additional modifications of the carbonized foam enhance its robustness. This is also proved by Stolz [54] and Chen [56] research groups. However, the carbonized MA foam introduced by Chen et al. [56] possessed great elasticity and was able to sustain large bending and strain compressions. In detail, the carbonized foam presented anisotropic response according to the compression curves, but all directions have excellent elasticity and compression strength of 10 KPa. Since elasticity of sponges is related to slenderness ratio (SR), the ratio between the fiber’s statistical average length between two junctions ($L_{f}^{-}$) and the cross section of the triangle shape ($L_{SS}^{-}$) of the sponge, an SR value of 26.7 for carbonized MA foam of 5 mg/cm^3^ indicates highest flexibility and elasticity. High SR value is also assisted with low density, high porosity (99%) and low carbon content (~9%). Low carbon yield is mainly because MA foam is rich in nitrogen content, which constitutes 50.9% of its structure, under pyrolysis nitrogen atoms are released as gases, causing the formation of larger pores, hence, reduce the brittleness of the foam, and increase its elasticity. Therefore, carbonization approach is not feasible to be applied for carbon and phenolic rich foams as they yield high carbon content causing the foams to be very brittle [110]. Indeed, carbonization of PU foam is not promising as in the case of MA since its carbon yield is extremely low to become a carbon foam due to the rich oxygen content in its structure. However, the carbonized foams suffer from poor recyclability as it loses 50% of its weight only after 10 absorption/burning cycles. Similar to Chen’s findings, the carbonized foams by Stolz’s group [55] (300–800 °C) suffered brittleness as well. In detail, when the samples (500–600 °C) underwent compression-decompression cycles at 50%, the thickness was reduced by 10%, and at 80% the loss was up to 20%. In addition, after 100 cycles of 80% compression, the loss in thickness involved 38% of the foam. This is because the carbonization has significantly reduced the mechanical resistance of the foam fibers, resulting in fiber fracture that are submitted to the direction of the stress and bending. Therefore, it can be concluded that elevated thermal treatment results in high flexibility and great brittleness. However, elastic recovery of the mechanically compressed sponges is determined using the Equation (8) [111]:

(8) $$\mathrm{Recovery}\;\%=\frac{t_{f}-{t\;}_{\mathrm{load}}}{t_{i}-t_{\mathrm{load}}\;}x100\;$$

where t_i_ and t_f_ are the respective initial and final thickness of the sponges after ‘n’ cycles of compression, t_load_ is the thickness under the load at certain strain %.

Notable research is done on nanoparticles incorporated foams, the usage of which is limited because of required substrate with strong adhesion (to prevent their aggregation), though. Liu et al. [112] fabricated durable and recyclable silica NPs and trimethoxysilane (DTMS) modified MA foams. The foam proved great adhesion stability, as it did not show any change in its skeleton after 100 sequence of adsorption/squeezing cycles using pump oil. Along with chemical stability, physical stability was also tested through rubbing the foam by sandpaper for 10 min. In addition, the foam remained unchanged after 100 cycles of absorption using DMF. The fabricated foam proved durability in seawater, extreme acidic solutions (pH = 1) and basic solutions (pH = 13) and its WCA was greater than 150°. Ren et al. [113] also achieved high durability when MA foam is dip coated in polyolefin containing chlorinated polypropylene (CPP), polypropylene (PP) and polyethylene (PE). The oil retention of all the modified foam was 80% over 100 cycles and it was even higher for the PP-MA over 1000 cycle of chloroform and diesel oil in which the foam retained 80% of its performance. The sponges were addressed by good mechanical stability through recovering to their original shape over 100 cycles at 70% compression test. Pristine MA foam is fragile in its nature; and hence, it is very crucial for modified foams to be used in oil cleanup to have high strain values to be able to stretch appropriately. The stress strain curves of the dip coated sponges did not indicate significant increment in the values compared to the pristine MA (104 ± 18 kPa), however, the strain values greatly increased to 17.7 ± 5.5% for the PP-MA and 32.2 ± 8.6% for CPP-MA compared to pristine strain (9.4 ± 1.5%). However, differently treated polymeric foams (MA and PU) with highly mechanical and chemical stability are reported in the literature and summarized in Table 2. 

## 4. Challenges and Possibilities of Commercialization

**Figure 17:**
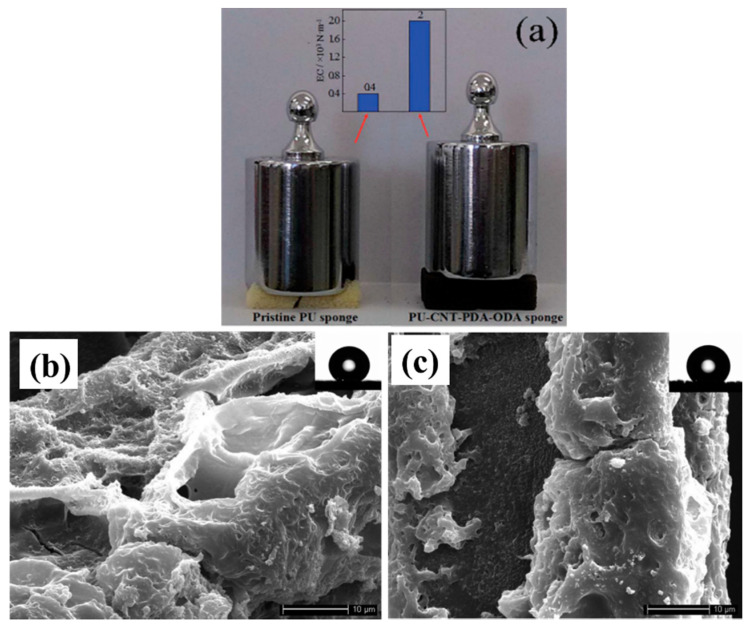
(**a**) Photoshoot for the comparison of elasticity between pristine PU foam and CNT/PDA/ODA PU sponge, and the SEM images of methyltrichlorosilane modified PU [115]; reproduced with permission from the American Chemical Society. (**b**) 300 cycles and (**c**) 400 cycles of oil/water separation [114]; reproduced with permission from the American Chemical Society.

Foams have gained great interest in emulsion separation recently because of their properties such as the low coast, high porosity, and great absorbance capacity. However, there are some challenges in the way of commercializing the sorbent-foams. First, the sponges can absorb different liquids including, water and oils, hence, their wettability should be developed towards selecting only one type of a liquid. This can be done through different methods and techniques such as dip coating [126], solution immersion [127], sugar templating, vapor phase polymerization [105] etc. and is developing in a faster rate. Although a number of reported sponges can withstand compression cycles, polymeric foams cannot withstand site applications such as bending and twisting [128]. Another challenge is that either using organic agent or organic-inorganic agents for the post modification of commercially available foams, different limitations can occur [129]. For instance, post modification via using organic agents as polymers and etching materials [63,130], can turn the polymeric foams into superhydrophobic foams by lowering the surface energy of the foam and increasing its roughness but the increase in roughness is random. In other words, the roughness is not uniformly distributed in the foam, which causes differences in the hydrophobicity areas within the foam itself. Indeed, the mechanical robustness of the organic treated sponge is a concern because after numerous oil sorption and desorption cycles, the sponge can lose its functionality gradually due to the low robustness of the original sponge and cannot be eliminated by simple organic layer on its surface. Post modification via organic/inorganic agents is efficient in the terms of durability and mechanical robustness [129,131,132,133]. The creation of secondary nanostructure on the foam can increase the roughness of the foam by creating bumpy surface on the foam and the organic materials works on binding the nanomaterial onto skeleton of the sponge. However, with multiple oil absorption/squeezing cycles the nanoparticles may rip off from the surface of the foam, which devalue the performance of the sponge. In addition, modification via nanomaterials is usually costly and require complex steps. Indeed, the absorption capacity and the flexibility of the organic/inorganic modified sponges are lowered. Moreover, the diameter of the pores and their intensity cannot be precisely controlled, and this type of modification and elasticity associated with the available commercial foams is greatly reduced (Figure 17a) [114,115]. Moreover, at high loading, the nanomaterials aggregate and initiate cracks in foams [114] as shown in Figure 17b,c.

## 5. Conclusions

The review summarizes and evaluates various protocols for an efficient modification of MA and PU foams and their efficiency towards separating both oil in water and water in oil mixtures. 

The motivation for this work is given by high potential of both types of foams due to their easy availability and low price, high porosity, and excellent sorption ability for both oil and water phase. The efficient separation ability of oil in water or water in oil require additional modifications of the foam’s wettability. Whereas a separation of oil in water emulsions need an enhancement of hydrophobicity and maintaining or even improvement oleophilicity, the separation of water in oil requires opposite surface behavior. Both strategies have been describing in a large number of papers, and this review aims to summarize and evaluate the most recent findings solely focused on the oil in water emulsions. Because the technical issues associated with a separation of stabilized emulsions, particularly with droplets size below 5 μm are very critical in water treatment and reuse, highly advanced and efficient methods are of much significance to investigate. This review critically addresses how the different kinds of surface modifications or functionalization methods tune the oil absorption and separation capability. Based on the thorough study, the following conclusions can be drawn: (i) surface modification by different active agents or surfactants regulate the wettability of the foams and thus helps in achieving superhydrophobicity and superoleophilicity, (ii) several nanoparticles by means of physical or chemical interactions also tune the surface properties of the foams, provided the secondary pollution by leaching of nanoparticles should be eliminated, (iii) both oil in water and water in oil emulsions require similar characteristics, however depending on the wettability, the performance of separation can be varied, (iv) major parameters that determine the oil/water separation efficiency of the foam include its separation efficiency, recyclability and mechanical/chemical/thermal durability over time. Though several advanced foams with outstanding separation efficiency have been already developed, the selective separation of oils is still a challenging research area, particularly regarding separation of surfactant stabilized emulsions with small droplets size (below 5 μm). 

## Figures and Tables

**Table 1:** Modified polymeric foams (MA and PU) developed for oil in water (OW) and water in oil (WO) emulsion separation. Treating material, and the available data about water contact angle, adsorption capacity (AC) (times the foam’s own weight), separation efficiency and additional information related to the foams or separation process are included.

Foam Type	Treatment	WCA	AC	Effeiciny (%)	Emulsion Type: Oil in Water (OWE) or Water in Oil (WOE)	Additional Information	Ref.
MA	Polybenzoxazine	162°	170	99.96%	Surfactant stabilized WOE	-	[89]
MA	rGO	164°	2010/5647 (mg/g)	97 ± 6% &95 ± 3%	OWE (crude oil)	Ratio: 10,000/30,000 (mg/l)	[90]
MA	β-FeOOH nanoparticles	155°	65–136	99.5%	OWE (pump oil)	1:25 (V_water_/V_oil_)	[91]
MA	caffeic acid-polyethyleneimine	150°	-	97%	SOWE	-	[92]
MA	dopamine (DA) and polyethyleneimine (PEI)	141. 9°	67.2–178.6	93.5%	OWE	-	[93]
MA	Mg(OH)_2_	160°	70–190	99.7%	OWE (toluene)	-	[94]
MA	Co-ZIF-L	-	26–61	97.7%	OWE (dodcane)	Ratio:1: 1000 *w/w*	[95]
MA	PDA/dodecanethiol (DDT)	158°	45.2–98.6	76.6–93.8%	OWE	-	[96]
MA	PVDF-HFP+Fe_3_O_4_ NPs	~130°	29.2–43.73	-	WOE	Ratio: (1:10 *w/w*)	[97]
PU	Stearic acid	151°	17.4–41.6	80%	OWE (toluene)	Oil concentration of 10:1 (Vwater/Voil)	[98]
PU	3,5-bis(trifluoromethyl) benzenediazonium tosylate [ADT-(CF_3_)_2_]	168	40–75	99%	OWE	-	[85]
PU	1,3-oxazolidin/1,4-dioxane, stearoyl chloride, and NaHCO3	152	23	-	WOE (crude)	-	[99]
PU	carbon cloth (CC)/ZnO/SA	160	40–70	-	OWE (toluene)	-	[100]
PU	octadecyltrichlorosilane (OTS)	156	25	~97.7%	WOE (toluene)	-	[101]
PU	PDA/Fe_3_O_4_/Ag	156	22.9–52.0	96.4%	OWE (toluene)	-	[102]
PU	Graphene	151.8	-	99.91%	SOWE	-	[78]
PU	MCFO/RGO nanocomposite	165	39.8–131.4	99.8–99.9%	WOE+SOWE	-	[103]
PU	Dopamine+ fly ash, and dodecanethiol	161	34–47	93%	OWE	-	[82]
PU	HDPE/Fe_3_O_4_	155	40–75	98.2%	WOE+SOWE (toluene)	(1:9 *v*:*v*)	[104]
PU	thermoplastic PU (TPU) /SiO_2_	149	30	95%	WOE	10 to 80 v.v%.	[105]
MA	Carbonization followed by 1H, 1H, 2H, 2H-perfluorodecanethiol coating	158	17–43	-	OWE+SOWE (tetrachloromethane)	-	[106]

**Table 2:** Modified polymeric foams (MA and PU) with high robustness reported in the literature. Treating material, recyclability (manual absorption/squeezing cycles), water contact angle of the foam after last absorption/squeezing cycles, durability of the foam tested in different harsh environments, compressibility in cycles and additional information regarding the recyclability and durability.

Foam	Treatment	Recycle (Cycles)	WCA (°)	Durability	Compressibility	Additional Information	Ref.
PU	rGO	1000	-	-	1000 cycles/80%	Foam lost 10% of its absorption capacity after cycles 1000	[58]
MA	PDA	100	158.3	-	1000 cycles/60%	Foam absorb flammable oil and organic solvents	[47]
PU	methyltrichlorsilane/ hexane solution	300	<150	Stabile at different pH and temperatures	-	-	[114]
MA	dopamine and 1H,1H,2H,2H-perfluorodecanethiol	100	158.3	Flame Retardancy and chemical and mechanical robustness.	1000 cycles/60%	Tested using cyclohexane oil	[48]
PU	CNTs,dopamine, and octadecylamine	150	~143	The foam is stable at different pH and temperatures.	-	Oil absorption capacity Decreased after 150 cycles by 14% using lubricating oil	[115]
PU	chromic acid,TMCS and TEOS	200	-	-	200 cycles/80%	The capacity lowers after 200 cycles	[116]
PU	rGO	50	-	-	-	Cost of rGO/PU foam ˂ Cost of graphene and carbon nanotube aerogels	[117]
MA	Dual Silanized SiO_2_	20	-	-	25–50 cycles/80%	5% deformation for the foam. Efficiency 98.3% after 20 cycles.	[118]
MA	PDA and P2VP-b-PDMS- copolymer	10	-	Foam kept in dichloromethane for 24 h and WCA of 146°–150°	-	The foam has switchable pH hydrophobicity, and it is robust in corrosive media.	[119]
MA	Spiropyran methacrylate (SPMA) derivative	-	-	-	100 cycles/50%	The wettability of the foam is light responsive.	[120]
MA	MoS_2_	50	150	Stable at abrasion test of 20 cycles.	20 cycles/90%	Recyclability tested using cyclohexane and silicon oil.	[121]
MA	PDA/PDVB	20	-	Immersed in ethanol for 3 days and ramined unchanged.	250 cycles	Tested using toluene.	[122]
MA	PC-O/Fe_3_O_4_	50	149	Stable at different pH.	-	92% oil retention after 100 cycles	[123]
MA	diamond-like carbon/TiO_2_	35	-	Foam is flame retardant	-	Tested using DMF.	[124]
MA	Kaolinite/ GO	30	-	Stable at different pH and temperatures.	-	94% adsorption capacity retention for diesel oil after 30 cycles.	[125]

## Data Availability

No new data were created or analyzed in this study.
